# Partners of people on ART - a New Evaluation of the Risks (The PARTNER study): design and methods

**DOI:** 10.1186/1471-2458-12-296

**Published:** 2012-06-25

**Authors:** Alison Rodger, Tina Bruun, Matthew Weait, Pietro Vernazza, Simon Collins, Vicente Estrada, Jan Van Lunzen, Giulio Maria Corbelli, Fiona Lampe, Andrew Phillips, Jens Lundgren

**Affiliations:** 1 Research Department of Infection & Population Health, University College London, London, UK; 2 Copenhagen HIV Programme, University of Copenhagen & Rigshospitalet, Copenhagen, DK, UK; 3 School of Law, Birkbeck College, London, UK; 4 Cantonal Hospital, St. Gallen, Switzerland; 5 HIV i-Base, London, UK; 6 Hospital Clinico San Carlos, Madrid, Spain; 7 University Medical Center Hamburg-Eppendorf, Hamburg-Eppendorf, Germany; 8 European AIDS Treatment group, Bruxelles, Belgium

**Keywords:** Serodifferent, HIV, Transmission, Antiretroviral therapy

## Abstract

****Background**:**

It is known that being on antiretroviral therapy reduces the risk of HIV transmission through sex. However it remains unknown what the absolute level of risk of transmission is in a person on ART with most recent measured HIV plasma viral load<50 c/mL in the absence of condom use. There are no data on risk of transmission for anal sex in MSM when the index partner is on ART.

****Methods/Design**:**

The PARTNER study is an international, observational multi-centre study, taking place from 2010 to 2014 in which HIV serodifferent partnerships who at enrolment reported recently having had condom-less vaginal or anal sexual intercourse are followed over time, with 46 monthly reporting of transmission risk behaviour through a confidential self completed risk behaviour questionnaire and with 46 monthly HIV testing for the HIV negative partner. The objective is to study (i) the risk of HIV transmission to partners, in particular in partnerships that continue not to use condoms consistently and the HIV-positive partner is on therapy with a viral load<50 copies/mL and (ii) why some partnerships do not use condoms, to describe the proportion who begin to adopt consistent condom use, and factors associated with this. For any negative partner who becomes infected phylogenetic analysis will be used following anonymisation of the samples to assess if transmission had been from the HIV infected partner.

****Discussion**:**

This observational study will provide missing information on the absolute risk of HIV transmission for both vaginal and anal sex when the index case is on ART with a VL<50 copies/mL in the absence of condom use.

## **Background**

It is consistently reported that a proportion of people with diagnosed HIV do not always use a condom when having sexual intercourse with partners of negative or unknown HIV status. There are likely to be many reasons for this, and these reasons may have changed over time. Increasingly, one reason for not using condoms is likely to be due to the person being on antiretroviral therapy with the plasma viral load being<50 copies/mL, and statements on the likely reduced infectiousness of people in this situation have been issued [1,2].

The HPTN 052 randomised controlled trial demonstrated a reduction in HIV transmission risk in heterosexual serodifferent couples due to ART use of 96% [3]. However as the HPTN 052 trial was performed in a population where condom use was prevalent (96% of those on ART reported regular condom use), the results demonstrate the added benefit from ART in the context of extensive condom use . Therefore it remains unknown what the absolute level of risk of transmission is in a person on ART with most recent measured plasma viral load<50 c/mL in the absence of condom use, particularly amongst MSM. This is a key factor that will help to determine whether more widespread and earlier use of ART will result in a substantial reduction in HIV incidence and hence be cost-effective, and is critical information for counselling.

### **Research questions**

The PARTNER study set out to follow serodifferent partnerships who initially report recently having had condomless sexual intercourse in order to study (i) the risk of HIV transmission to partners, in particular in partnerships that continue not to use condoms consistently and the HIV-positive partner is on therapy with a viral load<50 copies/mL and (ii) why some partnerships do not use condoms, to describe the proportion who begin to adopt consistent condom use, and factors associated with this.

## **Methods/Design**

### **Study design and sample size**

PARTNER is an observational study in which HIV serodiscordant partnerships are followed over time, with 46 monthly reporting of transmission risk behaviour and HIV testing for the HIV negative partner. Enrollment of subjects will cease at end of 2013 and follow up will cease in mid 2014 with enrolled partnerships followed initially for 2years. The study is co-ordinated jointly between UCL and the Copenhagen HIV Programme (CHIP). The protocol (Version 1: 6th May 2010) and a manual of operations were completed. Patient information sheets and consent forms, clinical research forms and study questionnaires were developed and all study documents are available on line on the CHIP PARTNER study website in 11 different languages (http://www.cphiv.dk/PARTNER/StudyDocuments/tabid/440/Default.aspx).

The sample size requirement was greater for the aim of studying transmission than for the aim of studying the proportion of partnerships that adopt consistent condom use so sample size was based on that needed to study transmission. For studying HIV transmission, the primary aim is the estimation of the transmission rate in partnerships which are having condomless sex and where the HIV positive partner has viral load<50 copies/mL. If the true transmission rate in this situation is<1 per 1000 person years of condomless sex partnerships with viral load<50, then with 2000 person years of observation with viral load<50 there is an 85% chance that the upper 95% confidence limit (i.e. the upper bound on the transmission rate) for the transmission rate is<0.0044 (i.e. 1 per 227 person years of condomless sex). We estimated that for 90% of included person time the HIV positive partner will have viral load<50 c/mL. Thus we will need to accrue 2222 person years of follow-up in order to accrue 2000 person years where the viral load is<50 c/mL. A proportion of partnerships will start to use condoms for all sex (as they will continue to be advised to do) so they will not be contributing person time of condomless sex. What this proportion will be is unknown, but we assume that this will be the case for one third of follow-up. This means that we will require 3333 person years of follow-up in total. Initially, 1666 partnerships will be identified, with the intention to follow them for up to 2years. If for any reason a partnership chooses/is unable to contribute for the entire length of time, replacement will occur to achieve the overall target of 3,333 person-years. Follow-up for all partnerships ceases when this target has been meet.

### **Recruitment and study procedures**

Seventy two HIV clinics in the UK and throughout Europe are participating in the study (the number may expand further). Study sites are detailed on the PARTNER study website (http://www.cphiv.dk/PARTNER/tabid/406/Default.aspx). Any patient on ART (regardless of VL) who was seen regularly for HIV care at a clinic and who has a stable partner not known to be HIV-infected and with whom he/she has had condomless sex in the past 1month (during which period the HIV negative partner was aware of the HIV status of the HIV positive partner) is eligible for inclusion in the study together with his/her partner. Patients could enrol with more than one partner, either concurrently or sequentially. Partner informed consent included consent to provide a blood sample if they become infected for phylogenetic analysis, so that the virus could be compared with that of index patient.

Subjects can self refer to the study. Study publicity in the form of leaflets, posters, press releases and articles were circulated to MSM press and national organizations. Eligible patients are identified in recruiting clinics by asking patients about condomless sexual activity. Patients are clearly informed about the risks of transmission by having condomless sex. Patients who reported having an ongoing stable partner, who was not known to be HIV-infected, with whom they had condomless sex in the past month are asked if they would consider asking their partner to come to the clinic to be enrolled in a study with them. Patients and their partners were then informed that the study is aiming to estimate the risk that HIV is transmitted from one partner to the other. The need for consistent condom use to avoid transmission is emphasised. The importance of the partner committing themselves to attending the clinic every 4 to 6months to get tested for HIV (where possible a 4th generation test was used which also picks up HIV antigen and thus more sensitive to detecting recent infections before specific antibody is present), and to complete a sexual risk behaviour questionnaire was emphasised. If the patients and their partner both agree to take part they signed separate informed consents, which includes identification by name of the partner and also that the HIV negative partner was aware of the HIV positive status of their partner when they had condomless penetrative sex.

Both partners complete baseline questionnaires on risk behaviour and then complete further risk behaviour questionnaires at each 46 monthly follow up visit. The negative partner tests for HIV at baseline and then again at each 4 to 6 monthly visit. Viral load is measured in patients at least 4 to 6 monthly. At each 4 monthly visit patients are asked about penetrative sex with the partner. If at any time the partner is found to be HIV-infected they completed the risk behaviour questionnaire for the final time and study follow-up ceased.

### **Data collection**

Data is collected by online case-record-forms completed by clinic staff and self completed paper risk behaviour questionnaires from both HIV positive and HIV negative partners. Questionnaire completion is done in a private place by participants and when completed the questionnaire is placed in an envelope provided by staff and then sent to Copenhagen HIV Programme [CHIP] (the study co-ordinating centre) without being seen by clinic staff. Participants are informed that clinic staff will not see questionnaires.

All study forms contain the PARTNER study ID and date of birth of the participant. No other patient identifiable information is included. The clinic keep a local log linking participant name and date of birth with study ID. The case Report Forms (CRFs) and risk behaviour questionnaires include the following forms and these can be accessed online at the PARTNER study website.

1. Baseline risk behaviour in index patient (patient to complete) 3 versions (i) patient male, partner male (ii) patient male, partner female, (iii) patient female, partner male

2. Baseline risk behaviour in partner (patient to complete) 3 versions as above.

3. Baseline clinical and antiretroviral drug use status on index patient (clinician/nurse to complete)

4. Follow-up risk behaviour in index patient (patient to complete) 3 versions as above.

5. Follow-up risk behaviour in partner (patient to complete) 3 versions as above.

6. Follow-up clinical and antiretroviral drug use status on index patient (clinician/nurse to complete)

7. Partner infection form risk behaviour (to be completed by partner if partner becomes infected with HIV) 3 versions as above.

8. Partner infection form (to be completed by clinician/nurse if partner becomes infected)

### **Phylogenetic analysis if a transmission occurs**

For new infections, where possible, phylogenetic analysis is to be used following anonymisation of the samples to allow comparison of the HIV positive partners virus with that of the newly infected partner, although the specific partnership will not be identifiable. If the viruses are very different by more than a certain percent of 3^rd^ bases (e.g. 5%) it is to be concluded that transmission has not been from the HIV infected partner. The anomymization process means that partnerships cannot be told the results of the virus comparison.

### **Data analysis plan and eligibility for time periods (between HIV tests in HIV- partner) to be included in the analysis**

**Figure 1:**
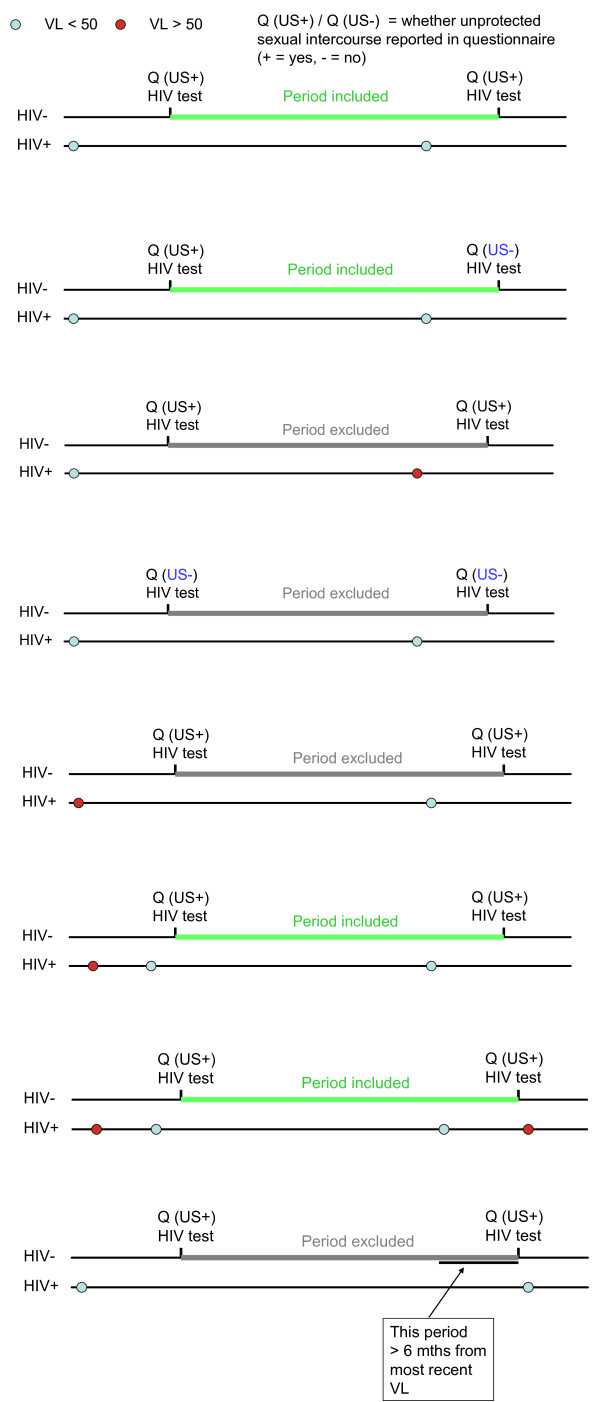
Examples of periods included and excluded for primary analysis.

We are assessing the proportion of partnerships that begin to adopt consistent condom use (i.e. reporting by both partners of 100% of episodes of sexual intercourse in which a condom was used) and factors associated with this using logistic regression. The primary analysis is estimation of the rate of infection in partners per person year of condomless sex partnership where the index patient has viral load <50 c/mL, excluding new infections that are shown to be phylogentically distinct from the HIV positive partners virus; i.e. transmission has not been from the HIV positive partner. This will be calculated as the number of infections identified at the end of eligible periods divided by the sum of the person time over eligible periods. A period was defined as the time between HIV tests in the HIV- partner. For a period to be eligible the following criteria had to be met: for all days in the period, the most recently measured viral load value in the HIV+partner must be<50 copies/mL and have been measured no more than 6months previously. In addition, a risk behaviour questionnaire, from the HIV negative partner, reporting condomless anal or vaginal sex together with the HIV+partner *within the period, or the previous period*, was available. The entire duration of a period fulfilling these criteria contributes to define the time spent at risk of transmission. Examples of periods eligible and not eligible are given in Figure 1.

In secondary analyses we will estimate (i) the rate of infection in partners *per condomless sex act* where index patient has viral load<50 c/mL, as opposed to per person year (this was done by summing numbers of acts of anal and vaginal intercourse over eligible periods) (ii) the rate of infection if we replace 50 copies/mL by 200 copies/mL in the above definition and (iii) the rate of transmission if we insist that the next viral load value in the HIV positive partner after the end of the period was also<50 copies/mL, (iv) the rate of transmission if we consider periods to be eligible if only oral sex was reported and (v) the rate of transmission if we ignor viral load measures made on the HIV positive partner which were within 4weeks of the end of the period (because of the lag time in obtaining a result).

### **Ethical committee review**

The study is conducted according to the Declaration of Helsinki in its current version (2004); the requirements of Good Clinical Practice (GCP) as defined in EU GCP Directive (2005/28/EC); Human Subject Protection and Data Protection Acts or with the local law and regulation, whichever affords greater protection of human subjects. Prior to the initiation of the study at each clinical research site, the protocol, all informed consent forms and the participant information materials are submitted to and approved by the sites Ethics Committee (IRB or IEC). In the UK, the study was reviewed and approved by the North West London REC 2 Ethics Committee (EC reference number 10/H0720/55). Ethics approval was obtained in-country for all other European sites involved in the study. In addition any future amendments to the study protocol will be submitted and approved by each sites Ethics Committee (IRB or IEC). After approval, sites registered for the protocol before screening potential participants, and must also register for any protocol amendments.

### **HIV transmission and risk of prosecution**

All countries participating in the study have, or have had, laws which potentially criminalize PLHIV. Such criminalization may be for transmission, exposure or non-disclosure, and for intentional, reckless or negligent behavior [4]. The laws used may be general (i.e. HIV is treated as a form of bodily harm), or HIV-specific. The offences may be found either in a countrys Criminal or Penal Code, or in its public health legislation. People who have been convicted of HIV-related offences are often sentenced to long periods of custodial punishment. The fact that PLHIV may be, or have in the past been, criminalized in countries participating in the study was a central concern in developing its methodology and ensuring its ethical approval. The confidentiality of all study participants was protected in accordance with GCP Guidelines and national regulations, and a review of national criminal and public health laws was undertaken. We recruited only in countries in which convictions resulting from unprotected sex or HIV transmission after disclosure of HIV-positive status had not occurred, and was judged very unlikely to ever occur in future. The key concern here was informed consent and whether this provided a defence to allegations of exposure or transmission in the participating countries. In some European countries consent is available as a defence only in very limited circumstances (in Norway, for example, it is available only where the partners concerned are effectively in a spousal relationship) and this precluded their inclusion in the study [5]. In contrast, some of the countries that are included are ones in which the law in this area has either been reformed or has suspended criminalization pending review. Thus, the Netherlands now only contemplates the prosecution of those who transmit HIV with malicious intent [6], while Denmark has suspended its HIV-specific offence until an analysis of its effectiveness has been completed [7]. It is important to note that patients and their partners were informed that the study was aiming to estimate the risk that HIV is transmitted from one partner to the other and why some partnerships do not use condoms, and factors associated with this. The need for consistent condom use to avoid transmission was emphasised at each contact. If the patients and their partner both agreed to take part they signed separate informed consents, which included identification by name and date of birth of the partner. All study participants signed all applicable approved informed consent forms prior to any study-related processes. The informed consent for HIV negative partners included explicit reference to the fact that their partner has HIV and there is transmission risk, particularly with unprotected sex. For all partnerships in which follow-up was discontinued (including those in which the reason for discontinuation of follow-up is infection of the HIV negative partner) identifiers such as study ID, clinic and day/month of birth, are deleted from the central database, thus anonymizing the data on the partnership. Thus, individuals who may have transmitted HIV to their partners could not subsequently be identified through the central study database. Should a negative partner have become infected with HIV during the study the analysis comparing the HIV positive partners virus with that of the newly infected partner, is done only after anonymization, and hence not linkable to the specific partnership.

## **Discussion**

With the publication of the results of the HPTN 052 trial there is now very strong evidence that virally suppressive ART reduces infectiousness of people with HIV through heterosexual sex [2,3,8-12]. However, there is insufficient data for transmission rates on ART in the absence of condom use in heterosexuals and no data for rates of transmission through anal sex in MSM which are likely to be different than rates for vaginal sex. Ongoing observational studies such as the PARTNER study will provide missing information in several key areas. Firstly, to more precisely estimate the absolute risk of HIV transmission using ART alone in sero-different couples having condom-less vaginal sex with suppressed VL on ART and to provide data on rates of transmission for anal sex in serodifferent couples (including MSM) which are likely to be different to vaginal sex.

## Abbreviations

HIV = Human Immunodeficiency Virus; ART = Antiretroviral therapy; HPTN = HIV Prevention Treatment Network; VL = Viral load; The PARTNER study = Partners of people on ART - a New Evaluation of the Risks; UCL = University College London; CHIP = Copenhagen HIV Programme; c/mL = Copies per millilitre; GCP = Good Clinical Practice; IRB = Institutional review board; IEC = Institutional ethics committees; ID = Identification.

## Competing interests

The authors declare that they have no competing interests.

## Authors' contributions

AR, SC, JL and AP conceived the study and obtained funding from the NIHR. AR, TB, JL, SC, MW and AP designed the study. AR drafted the manuscript with input from all authors. All authors read and approved the final manuscript.

## Funding

The PARTNER study represents independent research commissioned by the National Institute for Health Research (NIHR) under its Programme Grants for Applied Research funding scheme (RP-PG-0608-10142).

## Pre-publication history

The pre-publication history for this paper can be accessed here:

http://www.biomedcentral.com/1471-2458/12/296/prepub
